# Development of a Decision Aid for Patients With Low‐Risk Thyroid Cancer: A Mixed‐Methods Analysis of Feedback From Both Patient and Clinicians

**DOI:** 10.1002/wjs.70064

**Published:** 2025-08-30

**Authors:** Christine J. O'Neill, Ahmad Alam, Michelle Chapman, Melissa Carlson, Suzanne Clark‐Pitrolo, Elizabeth A. Fradgley, Christine Paul, Nicholas Zdenkowski, Christopher W. Rowe

**Affiliations:** ^1^ Surgical Services John Hunter Hospital Newcastle New South Wales Australia; ^2^ University of Newcastle Newcastle New South Wales Australia; ^3^ Hunter Medical Research Institute Newcastle New South Wales Australia; ^4^ Department of Endocrinology John Hunter Hospital Newcastle New South Wales Australia

**Keywords:** cancer survivorship, fear of cancer recurrence, shared decision making, thyroid cancer, thyroidectomy

## Abstract

**Introduction:**

Guideline‐driven de‐escalation of the extent of surgery for low‐risk thyroid cancer has made treatment decisions more complex. Shared decision‐making (SDM) is more involved than informed consent, improves patient satisfaction, and is considered standard of care. Patient decision aids (DA) can facilitate SDM but appropriate resources are lacking.

**Methods:**

DA development occurred in 3 main phases. First, a prototype DA was developed and refined by a working group (clinicians, behavioral scientists, nurses, and trained consumer). Nationwide clinician consultation sessions obtained mixed‐methods feedback leading to a hybrid paper‐web DA ready for patient testing. Second, the paper DA was used within clinically appropriate consultations (Bethesda 3–6 thyroid nodules) and patient feedback obtained with the Ottawa acceptability and decisional conflict scales. Three cycles of iterative changes were made to the DA. Patient focus groups led to further refinements. Third, 40 clinicians were invited to review DA materials, providing mixed‐methods feedback.

**Results:**

Initial clinician consultation sessions (*n* = 113) revealed that surgeons used information resources more frequently in, and were more satisfied with, their current patient discussions around thyroid cancer management compared with endocrinologists (88% vs. 32% and 95% vs. 46% respectively, *p* < 0.01 for both). 95% of clinicians were open to using the DA, but concerns regarding availability, appropriateness, flexibility, credibility and potential to lengthen consultations, were raised. Patients reported that the DA was useful (97% paper, 100% web) and sufficient (85% paper, 100% web). Decisional conflict was low (17 paper vs. 12 web). Qualitative feedback led to changes to improve visual appeal, readability and minimize emotive responses. Clinician review of DA (60% response) reported no bias (73% paper, 79% web) and 86% felt the DA would be easily incorporated into practice.

**Conclusion:**

We present a hybrid paper and web‐DA ready for wider testing in patients with low‐risk thyroid cancer to complement SDM regarding the extent of surgery.

## Graphical abstract

Guideline‐driven de‐escalation of the extent of surgery for low‐risk thyroid cancer has made treatment decisions more complex. Shared decision‐making (SDM) is more involved than informed consent, can improve patient satisfaction, and is considered standard of care. Patient decision aids (DA) can facilitate SDM but appropriate resources are lacking.

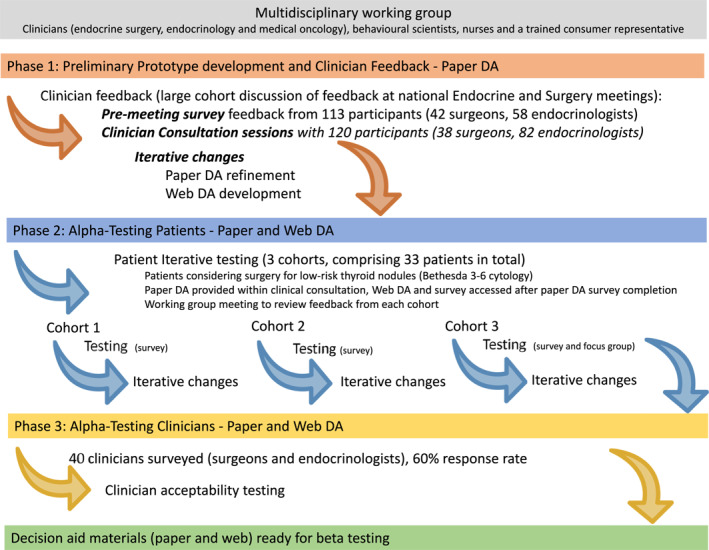

## 1 Introduction

Well differentiated thyroid cancer (WDTC) diagnoses have increased, largely due detection on imaging [1]. It is estimated that up to 70% of WDTC diagnoses in Australia may represent “overdiagnosis”, where incidental WDTC are clinically indolent, leaving patients at risk of “overtreatment”, and yet burdened with the psychosocial impacts of a “cancer diagnosis” [2, 3, 4]. With this in mind, and with confirmation of no survival advantage of total thyroidectomy (TT), for those with low‐risk WDTC [5], recent guidelines have advocated a de‐escalated approach to thyroid cancer surgery, namely hemithyroidectomy (HT), or active surveillance (AS) without surgery [6]. Despite this, TT remains the predominant procedure for those with low‐risk WDTC [7]. There is a need to investigate patient and surgeon preferences and values as they relate to decisions regarding surgery for WDTC [8, 9, 10, 11].

Shared‐decision‐making (SDM) involves, at a minimum, describing treatment options, tailoring information to individual patients, and creating choice awareness [12]. SDM is more involved than informed consent, and is increasingly seen as standard of care for healthcare decisions [13, 14]. Patient decision aids (DA) are one method of assisting SDM by providing credible and relevant information for patients, assisting them to contextualize and elucidate their own preferences. DAs can be presented via paper, web, video, audio, or in combination. Development should adhere to International Patient Decision Aid Standards (IPDAS) guidelines [15, 16], involving initial scoping with prototype development, “alpha” testing with iterative feedback from both patients and clinicians, and then “beta” field testing in “real life” conditions with consideration to barriers and facilitators for implementation [15, 17, 18].

Given the increased complexity of decision‐making in WDTC, there is a need to encourage and evaluate SDM in clinical practice. DAs are relatively new to thyroid cancer management with only a few being developed and evaluated world‐wide [19, 20, 21, 22]. There is no DA available to assist SDM regarding the extent of surgery for low‐risk WDTC in an Australian context. This study documents the development of a DA to fill this clinical need, following IPDAS principles (including prototype development and “alpha” testing). A DA ready for “beta” testing in clinical practice is presented.

## 2 Methods

**FIGURE 1:**
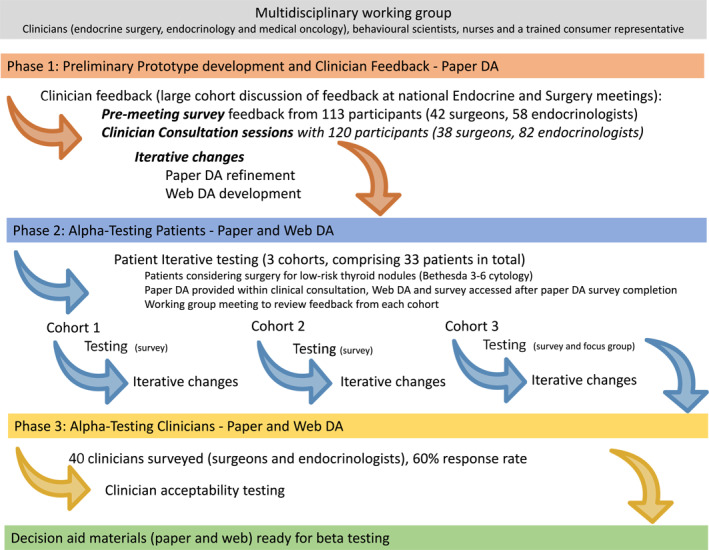
Summary of the decision aid development process including prototype creation and alpha testing in accordance with International Patient Decision Aid Standards (IPDAS).

DA development occurred in 3 phases [1]: prototype development and refinement [2]; alpha‐testing patients; and [3] alpha‐testing clinicians, Figure 1. A working group was formed including clinicians (endocrine surgery, endocrinology, and medical oncology), behavioral scientists, nurses and a trained consumer representative. The group developed a prototype paper‐DA based on current best evidence, guidelines, and expert opinion.

### 2.1 Phase 1: Preliminary Prototype Development and Clinician Feedback

The initial prototype paper‐DA was presented within consultation sessions during the 2022 annual surgical (Royal Australasian College of Surgeons Annual Scientific Congress Endocrine Surgery Section) and endocrinology (Endocrine Surgery of Australia Annual Seminar) meetings. A pre‐meeting survey was conducted (email invitation with Research Electronic Data Capture (REDCap) [23] weblink, via specialist societies, or QR‐code link within specialist meetings). The survey did not contain the prototype DA, but aimed to capture clinician demographics, current practice patterns, and perceptions around SDM (Supporting Information S1). Additional questions explored what an “ideal DA for low‐risk WDTC” would include, and how it would be implemented and the anticipated effect on clinical practice. Face‐to‐face clinician consultation sessions were facilitated by clinicians (CO and CR) and discussed both content and format of the DA, and explored logistical, patient, and clinician barriers (and mitigation strategies).

### 2.2 Phase 2: Alpha Testing—Patients

Patients aged > 18 years referred to clinicians (members of a mixed metropolitan and regional thyroid cancer multidisciplinary team) to discuss surgery for thyroid nodules with some suspicion of low‐risk WDTC (according the American Thyroid Association risk prediction) [6] were invited to participate. This included pre‐operative patients with thyroid nodules with Bethesda 3, 4, 5, or 6 cytology and those with a diagnosis post‐lobectomy of WDTC. Exclusion criteria included the inability to comprehend DA material or complete surveys (including language barriers, disability, or lack of computer access), or a likely diagnosis of intermediate or high‐risk thyroid cancer based on sonographic or clinical evidence of nodal metastases or extra‐thyroidal extension. The paper‐DA was introduced to the patient within the clinical consultation by the treating surgeon or endocrinologist. Clinicians were supplied with prompts to assist with introduction of the DA and discussion of survey completion. Patients accessed the survey via a QR code on the paper‐DA, Supporting Information S2. Upon completion of the survey regarding the paper‐DA, patients were directed to the web‐DA and invited to complete another survey (specific to the web‐DA), Supporting Information S3. Surveys collected demographic data, Ottawa acceptability scale [24], and decisional conflict scale [25, 26]. Open‐ended questions and free‐text boxes were included for participants to provide unstructured feedback. Patients who completed both surveys received a $30AUD gift voucher.

Iterative changes were undertaken following a full working group meeting to discuss feedback after 6–10 patient responses in each of three cycles. Feedback was incorporated into the DA and the new version disseminated prior to the next round of iterative feedback. At the conclusion of the iterative phase, patients who had taken part in the study or had been previously diagnosed with low‐risk WDTC (outside of the study period) were invited to focus groups. These were recorded and transcribed verbatim; codebook thematic analysis of responses was undertaken.

### 2.3 Phase 3: Alpha‐Testing Clinicians

Forty Australian‐based surgeons (endocrine, general, and otolaryngology, head and neck) and endocrinologists were sent the DA materials (updated after final patient feedback) and invited to complete a survey, Supporting Information S4. Clinicians included those who had indicated interest in Phase 1, with additional clinicians selected to ensure diversity of age, gender, practice location (metropolitan/regional), and type (public/private). The survey was administered via email containing a weblink to a REDCap Survey and collected demographic data, as well as the Ottawa acceptability scale [24]. Additional unstructured feedback was sought via open text boxes. Minor changes were made to finalize the DA to progress to beta clinical evaluation.

The study protocol was approved by the Hunter New England Human Research Ethics Committee (2023/ETH00819) and all patient participants provided informed consent. Quantitative data were stored in a REDCap database and qualitative data stored and analyzed using NVivo (QSR International, Melbourne, Australia). Surgeon and endocrinologist responses to the first survey were compared using Fisher's exact or Chi‐squared tests as appropriate.

## 3 Results

### 3.1 Phase 1: Mixed‐Methods Clinician Feedback of Prototype DA

Surveys prior to clinician consultation sessions (prior to introduction of the prototype DA) had 113 respondents including 42 surgeons (63% male), 58 endocrinologists (47% male), and 13 specialists in training (12 endocrinology, 61% female). Compared to endocrinologists, surgeons were more senior (71% vs. 44% in consultant practice > 10 years), more likely to attend a thyroid cancer MDT (80% vs. 45%), but less likely to be only in public hospital practice (5% vs. 21%). Four (9%) endocrinologists reported seeing more than 10 new thyroid cancer patients prior to surgery annually, whereas 68% of surgeons reported seeing more than 20 new thyroid cancer patients annually.

Survey results, comparing responses from surgeons and endocrinologists, are presented in Table 1. Additional information resources (picture, written or online) were used within consultations by 88% of surgeons compared with 32% of endocrinologists. Most clinicians felt patients benefited from involvement in treatment decisions (63% surgeons and 84% endocrinologists), reporting that they actively involve patients in decision‐making (79% surgeons and 71% of endocrinologists). Most (95%) surgeons were satisfied with their discussion around treatment options for low‐risk WDTC compared with 49% of endocrinologists, *p* < 0.01. Two (2%) respondents anticipated they would not use a DA, 67% would use it in all relevant clinical scenarios and 31% would use it selectively. There was a clear preference for the DA to be introduced to the patient by a specialist either within or following a consultation, as well as a preference (52%) for both paper and web‐based materials.

**TABLE 1:** Survey of clinician perceptions regarding shared decision‐making (SDM) and the clinical utility and format of a decision aid (DA) prior to review of any DA materials.

	Surgeon *n* = 42^a^	Endocrinologist *n* = 58^a^	
Current use of information resources^b^	*n* = 40	*n* = 53	*p* < 0.01^c^
Nil	5 (13%)	36 (68%)	
Draw picture	21 (53%)	12 (23%)	
Own written material	12 (30%)	4 (8%)	
Written material form other source	13 (33%)	8 (15%)	
Website/other information	4 (10%)	6 (11%)	
Current approach to SDM	*n* = 38	*n* = 52	*p* = 0.65
Clinician recommended treatment plan	8 (21%)	15 (29%)	
Plan equally shared	19 (50%)	25 (48%)	
Patient given options and they decide	11 (29%)	12 (23%)	
Current satisfaction with discussion around extent of surgery for low‐risk DTC	*n* = 38	*n* = 50	*p* < 0.01
Very satisfied, no improvements required	15 (39%)	2 (4%)	
Somewhat satisfied, could be improved	21 (55%)	21 (42%)	
It's OK, I would like to make changes	2 (5%)	19 (38%)	
This is an area I would like to improve	0	8 (16%)	
Areas of improvement^b^	*n* = 38	*n* = 50	
The discussion is too long/complex	3 (8%)	6 (12%)	
Clearer explanation of treatment options	5 (13%)	24 (48%)	
Clearer explanation/documentation of risks	7 (18%)	32 (64%)	
I Would like to involve patient more in SDM	7 (18%)	15 (30%)	
Patient benefit from involvement in treatment decisions?	*n* = 38	*n* = 52	*p* = 0.02
Yes	24 (63%)	44 (84%)	
No/not sure	14 (37%)	8 (15%)	
Anticipated clinician use of DA	*n* = 37	*n* = 50	*p* = 0.49
In all consultations where relevant	22 (59%)	36 (72%)	
Selectively	14 (38%)	13 (26%)	
Rarely/not at all	1 (3%)	1 (2%)	
Timing of introduction of DA to patient^b^	*n* = 37	*n* = 50	*p* = 0.74
Before specialist consultation	5 (14%)	11 (22%)	
During specialist consultation	28 (76%)	41 (82%)	
Between two specialist consultations	14 (38%)	19 (38%)	
Preferred DA format	*n* = 40	*n* = 57	*p* = 0.01
Paper only	10 (25%)	7 (12%)	
Electronic only (web or app)	6 (15%)	24 (42%)	
Paper and electronic	24 (60%)	26 (46%)	
Predicted impact of DA on consultation time	*n* = 37	*n* = 50	*p* = 0.13
Shorten	3 (8%)	11 (22%)	
No effect	24 (65%)	23 (46%)	
Lengthen	10 (27%)	16 (32%)	
Perceived barriers to DA use^b^	*n* = 37	*n* = 50	
Availability for clinicians	23 (62%)	41 (82%)	
Availability for patients	17 (46%)	21 (42%)	
Culture of doctor‐drive decision‐making	8 (22%)	6 (12%)	
Concern about excess information	10 (27%)	11 (22%)	
Clinical concern about overwhelming patient	12 (32%)	20 (40%)	
Patient reluctance to participate in decisions	9 (24%)	12 (24%)	
Lack of applicability to specific scenarios	16 (43%)	16 (32%)	
Lack of credibility of such tools	7 (19%)	13 (26%)	

^a^ Forced responses were not used, the number of respondents to each question is listed at each item.

^b^ Multiple options could be selected, Chi Square not performed.

^c^ Fisher's exact (nil resources vs. use of resources, surgeons vs. endocrinologists).

The first prototype DA presented to clinician consultation sessions included only an A4 size, double‐sided, single page designed for use within a clinical consultation (feedback summarized in Table 2). There was general support for a simple paper‐based tool, but clinicians expressed the need for information to be credible, patient‐centric, flexible, and simply presented such that it could be easily incorporated into consultations, without increasing the length or complexity of the clinician–patient encounter. It was acknowledged that not all information could be contained within a single page; there was strong support for a web‐based DA with more detailed information alongside the paper‐DA. Based on this feedback, a website was developed prior to patient testing.

**TABLE 2:** Clinician consultation session feedback on the DA.

Content development	Risk factors—age, genetics, clear definition of low risk
	Ability to individualize information based on tumor and patient characteristics
	Need for additional information beyond the paper DA
	Patient‐centric phrasing
Clinician factors relating to implementation	Address concerns regarding impact on the length of consultation
	Simple tool for use within a standard consultation
	Provide education and training on use of tool
	Demonstrate value of the tool
	Ensure credibility and trustworthiness of the tool
	Address concerns regarding litigation and hesitancy
	Address concerns around accessibility
	Address concerns regarding use of tool out of clinical context
Patient factors relating to implementation	Language—ensure clear, simple, informative language
	Adaptability to cultural norms regarding decision‐making
	Ability to translate in multiple languages
	Accessibility for patients without internet, cognitive or mental health concerns
Logistical factors relating to implementation	Tools simplicity valued
	Centralization of thyroid cancer care
	Lack of staff and time
	Complexity and cost of tool
	Accessibility and adaption to telehealth
Strategies to mitigate implementation barriers	Consider use of allied health or nursing staff
	Website available easily to patients but recommended by clinicians
	Follow solid development process
	Seek adequate exposure and endorsement for relevant professional societies and leaders in the field

Perceived barriers included concerns about DA availability for both patients and clinicians, excess information overwhelming the patient, patient reluctance to participate in SDM, increasing the length of the consultation, the credibility of the DA, and lack of applicability to specific clinical scenarios.

### 3.2 Phase 2: Alpha‐Testing Patients

**FIGURE 2:**
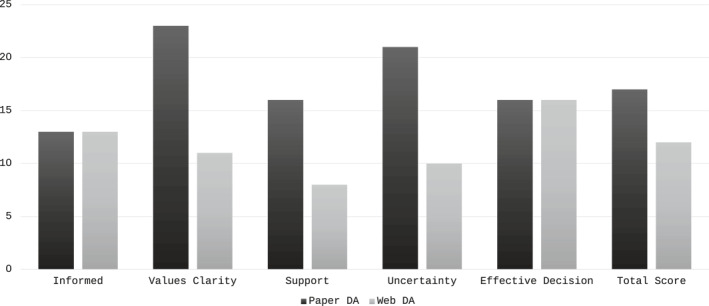
Decisional conflict scale scores during patient iterative testing phase. Subscale scores (0–100) are presented for each of the paper decision aid and web decision aid. Scores < 25 are associated with implementing decisions, scores > 37.5 are associated with decision delay or feeling unsure [24].

Across three phases of iterative testing, 33 patients responded to the survey of the paper‐DA and 22 to the web‐DA survey. Most were female (88%), from a metropolitan area (79%), and aged > 50 years (82%). The working group met on three occasions to review feedback and discuss changes. After each meeting, changes were made to the DA prior to the next phase of patient data collection. Low levels of decisional conflict were seen in the survey after review of the paper‐DA but these were lower again after review of the web‐DA, Figure 2. High levels of acceptability and low rates of bias were reported, Table 3.

**TABLE 3:** Patient acceptability of the decision aid.

	Paper decision aid	Web decision aid
	*N* = 33	*N* = 22
Information presentation		
Excellent	15 (46%)	13 (59%)
Good	16 (49%)	9 (41%)
Fair	2 (6%)	0
Treatment comparisons		
Excellent	15 (46%)	13 (59%)
Good	17 (52%)	9 (41%)
Fair	1 (3%)	0
Length of DA		
Too much	0	4 (18%)
Just right	31 (94%)	18 (82%)
Too little	2 (6%)	0
Amount of information		
Too much	0	2 (9%)
Just right	30 (91%)	20 (91%)
Too little	3 (9%)	0
Information favored		
Total thyroidectomy	3 (9%)	1 (5%)
Hemithyroidectomy	1 (3%)	1 (5%)
Active surveillance	1 (3%)	2 (5%)
Well balanced	28 (85%)	29 (86%)
Agreed information was useful?	32 (97%)	22 (100%)
Agreed information was sufficient?	28 (85%)	22 (100%)
When would you find this most useful?		
Before appointment	N/A^a^	2 (9%)
During appointment	7 (21%)	N/A^a^
After appointment	12 (37%)	5 (23%)
Both during and after appointment	16 (49%)	15 (63%)

*Note:* Patients were asked to complete the Ottawa acceptability scale for decision aids [24]. The paper aid contained a QR code linking the survey. At the completion of the survey regarding the paper‐DA, patients were directed to the website and the survey regarding the web‐DA.

^a^ N/A—the paper‐DA was introduced within an appointment so was not available prior. The option of viewing the web DA during the appointment was not given as an option.

Two focus groups were undertaken, one face‐to‐face (three participants) and one virtual (two participants). Codebook thematic analysis of the feedback centered around three themes: appropriateness, content and use, and clinical and patient context. It was acknowledged that there were many different pathways to the diagnosis of thyroid cancer, including post‐operative for some, and that information needed to be flexible and individualized. Credibility of the information, including having it supplied by a professional rather than freely available, was deemed preferable: “*having good information from a doctor is really helpful; everyone's got an opinion, there's lots of myths surrounding thyroids*”. Patients appreciated information that was centered on their questions and concerns rather than clinician‐based concerns.

Patients requested content changes in language to improve both readability and decrease fear associated with prior experiences of cancer: “*I know there's some medical words you can't get around… like recurrence and detected… maybe some plainer language”* and *“my kids have been without their dad for a proportion of their life, and that was from cancer… so even the fact that it’s got the word, low‐risk cancer, it's a little bit confronting*”. The introduction of visual representation of TT, HT and AS was well received. Clarification was required around the term “low‐risk”: *“so obviously I've got a low‐risk something or other, it's too small for them to find out what it is”.*

Patients reported that the paper‐DA was more useful if introduced and used within the context of a consultation: “*The doctor gave really good information and covered a lot of things but when I went home and I didn't have them there to ask questions I found this really helpful*”. Others reported that the aid was useful to convey information to their family members: “*my mother was terribly anxious, I took the tool and showed her, ‘look, it's less than a 1% chance’, so I found it quite useful for that*”.

### 3.3 Summary of Changes Made to the DA Following Patient Alpha‐Testing

Paper‐DA updates included reformatting and esthetic improvements, reworking graphics to visually represent surgical options, rewording to improve readability and reduce emotive responses, clarification around short and long‐term surgical risks, as well as the ability to “change your mind” about surgery if choosing AS.

There was clear consensus that the paper‐DA was sufficient for many and that additional information on the web could be sought voluntarily. Additions to the web‐DA included expanded information regarding recovery after surgery, the addition of a frequently asked questions section, and visual representation of the patient journey. Finally, the web‐DA integrated an interactive component, where patients could begin to test their informational understanding with real‐time feedback, formulate and record personal values and questions, and receive prompting to reflect on decisional readiness.

### 3.4 Phase 3: Alpha‐Testing Clinician

Survey responses were received from 24 clinicians (response rate 60%, 58% female, 67% < 50 years). Of these, 75% were surgeons, 74% based in a metropolitan area, 60% reported > 10 years of consultant practice, and 79% reported treating at least one new patient with WDTC per month. None felt the paper‐ or web‐DA would harm patients, but only 40% felt the paper‐DA would improve informed decision‐making for patients. Concerns were raised that introducing the paper‐DA within a consultation might increase its duration (29%) but most responding clinicians reported that they would consider using the paper‐DA: 36% with all relevant patients and 64% selectively, anticipating minimal change to current practice patterns.

The web‐DA survey received 19 clinician responses with universal agreement that it was suitable for helping patients make value‐based decisions. There were concerns that additional information may be unnecessary (43% paper, 38% web), create patient anxiety (36% paper 57% web), or confusion (50% paper, 57% web). It was also noted that language and literacy barriers may limit use of the aid in practice. Most felt the DA was well balanced without bias (73% paper, 79% web), but some felt there was bias toward active surveillance (14% paper, 11% web) or hemithyroidectomy (14% paper, 11% web). Several clinicians requested additional information be added to the paper‐DA regarding benign nodules, the possibility of an intermediate risk WDTC, individualized clinical features or risks of surgery, and follow‐up. Concerns were also raised regarding patient decision‐making styles (especially for those with deferential approaches who might prefer clinician‐led decision‐making) and utility of the web‐DA for those without internet skills or access. The working party reviewed all feedback, updating the DA ready for beta‐testing in clinical practice (trial underway, ACTRN112624001356550).

## 4 Discussion

**FIGURE 3:**
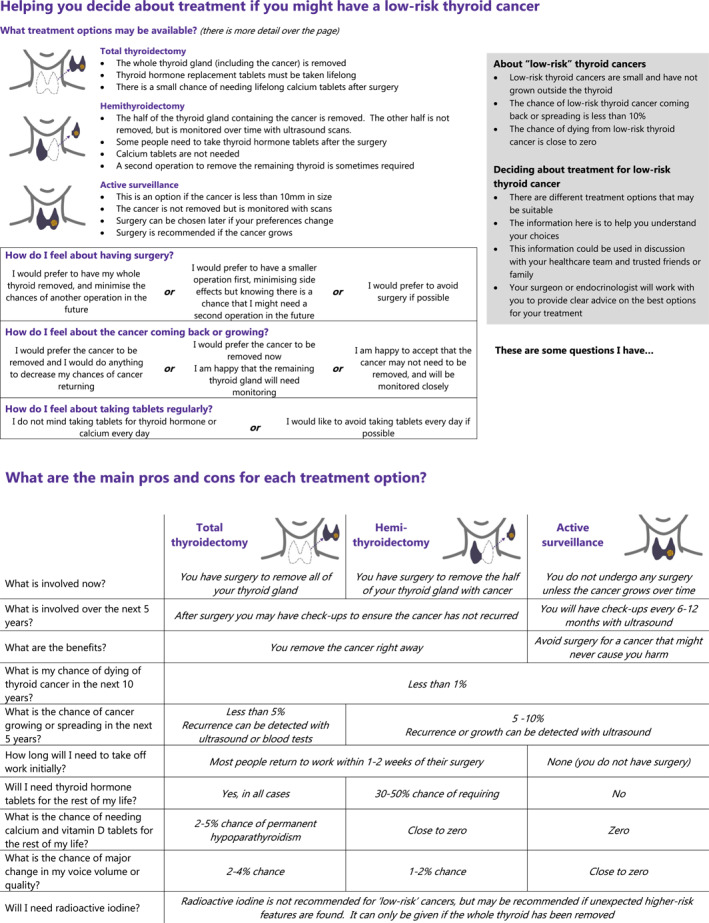
Paper decision aid: The revised, double‐sided paper decision aid presented here is designed to be introduced and used within a consultation and then taken home by the patient, also available as a downloadable at Supporting Information S5.

This manuscript documents the development of a hybrid paper‐ and web‐DA for low‐risk WDTC. Following IPDAS guidelines we have developed an initial prototype with clinician input, tested with mixed‐methods, made iterative changes in collaboration with patients, and returned to clinicians for finalization of information. The DA is now ready for beta testing within clinical practice (Figure 3, also available for download as a PDF at Supporting Information S5, and available at: https://thyroidology.au/thyroid_aid/). This development process has demonstrated that both clinicians and patients have a desire for credible information that is flexible to individualized clinical presentations. Clinicians are open to incorporating this DA into clinical practice.

Surgeons commonly use written information within consultations, particularly when obtaining informed consent [27]. The initial clinician survey confirmed that both published written information, bespoke drawings or annotated information sheets were commonly used by surgeons when discussing treatment options for low‐risk WDTC. Visual aids, or formal DAs (such as that presented here), go beyond the need to provide informed consent for a procedure, and have been shown to improve patient comprehension and knowledge, increase satisfaction and reduce anxiety [28]. Patient DAs have also been shown to decrease decisional conflict and longer term decisional regret, which may improve the quality of life [29, 30, 31, 32]. Although DAs are useful tools, they are designed to complement true SDM, which requires the clinician to partner with patients in decisions, empower them with information, elicit their values and preferences, and then share the treatment plan [33, 34, 35]. Despite SDM being widely recommended, there is discrepancy as its exact definition and measurement within clinical practice [12, 36, 37].

Key components of SDM include recognizing and acknowledging that a decision is required, knowing and understanding the best available clinical evidence, and incorporating the patient's values and preferences into that decision [34, 38]. To undertake SDM, clinicians need to be competent in their area of clinical practice, build rapport with the patient (and their families or support persons), be able to elicit patient preferences and values within their cultural context, provide appropriate, comprehensible information, and finally make shared decisions individualized to both the clinical situation and patient's personal context [39]. There are few studies examining SDM in WDTC, some utilizing hypothetical situations, via online surveys [40, 41, 42, 43, 44, 45] and others in clinical prospective studies [46, 47]. In these studies, SDM allowed patients to choose more conservative options for treatment. Information crucial to decision‐making included risks of cancer recurrence, further surgery, voice change, and thyroid hormone replacement [48, 49]. In addition, the emotional response to the term “cancer” has been suggested as a driver of decisions toward more extensive surgery [50, 51]. This needs to be acknowledged and addressed within clinical consultations.

Barriers to implementation of SDM in clinical practice include clinician anxiety around cancer recurrence or progression, and lack of skills in the process of SDM [11, 49, 52, 53]. Other important factors include both clinician and patient decision‐making and communication styles as well as health behavior preferences [54, 55, 56]. This study also highlights differences in surgeon and endocrinologist approaches to SDM in low‐risk thyroid cancer, suggesting that nuanced implementation strategies may be required (particularly around formatting, integration with current resource materials and perceived need for improvement). Patients express deep levels of trust and confidence in their clinicians and can derive significant psychosocial support from them, in addition to clinical care [57, 58]. Family and friends are also critical to decision‐making. In this study, patients utilized the DA to assist with conveying health information to family and support persons. Potential barriers identified by this study include clinician perception that SDM increases the duration of consultations, or increases patient confusion. A prospective study is required to determine whether these perceptions are accurate, or effect only a subset of patient–clinician dyads.

The process of development of this DA aimed to follow international guidelines and to provide opportunity for wide input. We acknowledge that the input received is subject to selection bias with clinicians and patients who are more open to discussion around SDM more likely to have responded to the surveys and added qualitative input. The patients recruited were from only one geographical area and included those who may not have been “ideal” recipients of the DA (those with Bethesda 3 or 4 nodules and those with a WDTC diagnosis following a diagnostic HT). This may have affected feedback received. Although during this process the paper DA was introduced by a clinician, we did not control how the clinician used the DA during the consultation and no feedback regarding communication of SDM within the consultation was received.

## 5 Conclusion

We present an IPDAS‐concordant alpha‐tested hybrid paper‐ and web‐DA ready for testing in clinical practice with a more diverse patient population with low‐risk WDTC, who are actively making treatment decisions. Further research is required to study the implementation and wider acceptability of the DA within clinical practice, as well as its impact on SDM, decisional conflict and regret, and health related quality of life.

## Author Contributions

**Christine J. O’Neill:** conceptualization, funding acquisition, writing – original draft, methodology, visualization, writing – review and editing, project administration, resources, supervision, investigation, formal analysis, data curation. **Ahmad Alam:** investigation, writing – review and editing, data curation, methodology. **Michelle Chapman:** writing – review and editing, validation, formal analysis, project administration, data curation, investigation. **Melissa Carlson:** methodology, writing – review and editing, formal analysis, supervision, data curation, investigation. **Suzanne Clark‐Pitrolo:** investigation, methodology, validation, writing – review and editing, data curation. **Elizabeth A. Fradgley:** conceptualization, investigation, methodology, supervision, writing – review and editing. **Christine Paul:** conceptualization, investigation, writing – review and editing, visualization, supervision, data curation. **Nicholas Zdenkowski:** conceptualization, writing – review and editing, methodology, formal analysis, supervision, investigation. **Christopher W. Rowe:** conceptualization, investigation, writing – review and editing, visualization, methodology, software, formal analysis, project administration, data curation, supervision, resources.

## Conflicts of Interest

The authors declare no conflicts of interest.

## Supporting information


Supporting information S1

Supporting Information S2

Supporting Information S3

Supporting Information S4

Supporting Information S5

## Data Availability

The data that support the findings of this study are available on request from the corresponding author. The data are not publicly available due to privacy or ethical restrictions.

## References

[1] L. Davies and H. G. Welch , “Current Thyroid Cancer Trends in the United States,” JAMA Otolaryngology–Head & Neck Surgery 140, no. 4 (2014): 317, 10.1001/jamaoto.2014.1. 24557566

[2] P. P. Glasziou , M. A. Jones , T. Pathirana , A. L. Barratt , and K. J. L. Bell , “Estimating the Magnitude of Cancer Overdiagnosis in Australia,” Medical Journal of Australia 212, no. 4 (2020): 163–168, 10.5694/mja2.50455. 31858624 PMC7065073

[3] M. Li , L. D. Maso , and S. Vaccarella , “Global Trends in Thyroid Cancer Incidence and the Impact of Overdiagnosis,” Lancet Diabetes & Endocrinology 8, no. 6 (2020): 468–470, 10.1016/s2213-8587(20)30115-7. 32445733

[4] S. Jegerlehner , J. L. Bulliard , D. Aujesky , et al., “Overdiagnosis and Overtreatment of Thyroid Cancer: A Population‐Based Temporal Trend Study,” PLoS One 12, no. 6 (2017): e0179387, 10.1371/journal.pone.0179387. 28614405 PMC5470703

[5] M. A. Adam , J. Pura , L. Gu , et al., “Extent of Surgery for Papillary Thyroid Cancer Is Not Associated With Survival,” Annals of Surgery 260, no. 4 (2014): 601–607, 10.1097/sla.0000000000000925. 25203876 PMC4532384

[6] B. R. Haugen , E. K. Alexander , K. C. Bible , et al., “2015 American Thyroid Association Management Guidelines for Adult Patients With Thyroid Nodules and Differentiated Thyroid Cancer: The American Thyroid Association Guidelines Task Force on Thyroid Nodules and Differentiated Thyroid Cancer,” Thyroid 26, no. 1 (2016): 1–133, 10.1089/thy.2015.0020. 26462967 PMC4739132

[7] E. Pasqual , J. A. Sosa , Y. Chen , S. J. Schonfeld , A. B. De Gonza , “Trends in the Management of Localized Papillary Thyroid,” Thyroid 32, no. 4 (2022): 1–14, 10.1089/thy.2021.0557. 35078347 PMC9048184

[8] A. M. Sawka , S. Ghai , L. Rotstein , et al., “A Quantitative Analysis Examining Patients’ Choice of Active Surveillance or Surgery for Managing Low Risk Papillary Thyroid Cancer,” Thyroid 32, no. 3 (2022): 1–25, 10.1089/thy.2021.0485. 35019770

[9] M. A. Schumm , M. L. Shu , A. M. Leung , et al., “Patient Preference in Physician Decision‐Making for Patients With Low‐ to Intermediate‐Risk Differentiated Thyroid Cancer,” JAMA Surgery 158, no. 8 (2023): 1–3: [Internet], 10.1001/jamasurg.2023.0359. PMC10157501 37133872

[10] W. Yang , Y. K. Lee , P. Lorgelly , S. N. Rogers , and D. Kim , “Challenges of Shared Decision‐Making by Clinicians and Patients With Low‐Risk Differentiated Thyroid Cancer: A Systematic Review and Meta‐Ethnography,” JAMA Otolaryngology–Head & Neck Surgery 149, no. 5 (2023): 452–459, 10.1001/jamaoto.2023.0101. 36951823

[11] A. S. Chiu , M. C. Saucke , K. Bushaw , et al., “The Relative Importance of Treatment Outcomes to Surgeons’ Recommendations for Low‐Risk Thyroid Cancer,” Surgery (United States) 173, no. 1 (2023): 183–188: [Internet], 10.1016/j.surg.2022.05.002. PMC12781660 36182602

[12] H. Bomhof‐Roordink , F. R. Gärtner , A. M. Stiggelbout , and A. H. Pieterse , “Key Components of Shared Decision Making Models: A Systematic Review,” BMJ Open 9, no. 12 (2019): e031763, 10.1136/bmjopen-2019-031763. PMC6937101 31852700

[13] C. J. Manta , J. Oritz , B. W. Moulton , and S. S. Sonnad , “From the Patient Perspective, Consent Forms Fall Short of Providing Information to Guide Decision Making,” Journal of Patient Safety 17, no. 3 (2021): e149–e154, 10.1097/pts.0000000000000310. 27490160 PMC5290300

[14] US Preventive Services Task Force , “Collaboration and Shared Decision‐Making Between Patients and Clinicians in Preventive Health Care Decisions and US Preventive Services Task Force Recommendations,” JAMA 327, no. 12 (March 2022): 1171–1176: [Internet], 10.1001/jama.2022.3267. 35315879

[15] A. Coulter , D. Stilwell , J. Kryworuchko , P. D. Mullen , C. J. Ng , and T. Van Der Weijden , “A Systematic Development Process for Patient Decision Aids,” supplement, BMC Medical Informatics and Decision Making 13, no. S2 (2013): S2, 10.1186/1472-6947-13-s2-s2. PMC4044159 24625093

[16] N. Joseph‐Williams , R. Newcombe , M. Politi , et al., “Toward Minimum Standards for Certifying Patient Decision Aids: A Modified Delphi Consensus Process,” Medical Decision Making 34, no. 6 (2014): 699–710, 10.1177/0272989x13501721. 23963501

[17] G. Elwyn , I. Scholl , C. Tietbohl , et al., “‘Many Miles to Go.’: A Systematic Review of the Implementation of Patient Decision Support Interventions into Routine Clinical Practice,” supplement, BMC Medical Informatics and Decision Making 13, no. S2 (2013): 1–10, 10.1186/1472-6947-13-s2-s14. 24625083 PMC4044318

[18] J. R. Covvey , K. M. Kamal , E. E. Gorse , et al., “Barriers and Facilitators to Shared Decision‐Making in Oncology: A Systematic Review of the Literature,” Supportive Care in Cancer 27, no. 5 (2019): 1613–1637, 10.1007/s00520-019-04675-7. 30737578

[19] S. C. Pitt and M. C. Saucke , “Novel Decision Support Interventions for Low‐Risk Thyroid Cancer,” JAMA Otolaryngology?Head & Neck Surgery 146, no. 11 (November 2020): 1079–1081, 10.1001/jamaoto.2020.2279. 32970109 PMC7516805

[20] A. M. Sawka , S. Straus , L. Rotstein , et al., “Randomized Controlled Trial of a Computerized Decision Aid on Adjuvant Radioactive Iodine Treatment for Patients With Early‐Stage Papillary Thyroid Cancer,” Journal of Clinical Oncology 30, no. 23 (2012): 2906–2911, 10.1200/jco.2011.41.2734. 22753906

[21] J. P. Brito , A. Castaneda‐Guarderas , M. R. Gionfriddo , et al., “Development and Pilot Testing of an Encounter Tool for Shared Decision Making About the Treatment of Graves’ Disease,” Thyroid 25, no. 11 (November 2015): 1191–1198, 10.1089/thy.2015.0277. 26413979 PMC4652182

[22] A. Koot , R. Hermens , P. Ottevanger , R. Netea‐Maier , and P. Stalmeier , “Patient Decision Aids for Patients With Differentiated Thyroid Carcinoma: Development Process and Alpha and Beta Testing,” Front Endocrinol (Lausanne) 14, no. May (2023): 1–11, 10.3389/fendo.2023.1162537. PMC10264809 37324263

[23] P. A. Harris , R. Taylor , R. Thielke , J. Payne , N. Gonzalez , and J. G. Conde , “Research Electronic Data Capture (REDCap)‐A Metadata‐Driven Methodology and Workflow Process for Providing Translational Research Informatics Support,” Journal of Biomedical Informatics 42, no. 2 (2009): 377–381, 10.1016/j.jbi.2008.08.010. 18929686 PMC2700030

[24] A. O’Connor and A. Cranney , User Manual—Acceptability (Ottawa Hospital Reserach Institute) [Internet], 1996 (updated 2022), http://decisionaid.ohri.ca/docs/develop/User_Manuals/UM_Acceptability.pdf.

[25] A. M. O’Connor , User Manual – Decisional Conflict Scale (1993), 1–16, https://decisionaid.ohri.ca/eval_dcs.html.

[26] A. M. O’Connor , “Validation of a Decisional Conflict Scale,” Medical Decision Making 15, no. 1 (1995): 25–30, 10.1177/0272989x9501500105. 7898294

[27] C. Kearns , N. Kearns , and A. M. Paisley , “The Art of Consent: Visual Materials Help Adult Patients Make Informed Choices About Surgical Care,” Journal of Visual Communication in Medicine 43, no. 2 (2020): 76–83, 10.1080/17453054.2019.1671168. 31799883

[28] S. M. Cohen , M. Baimas‐George , C. Ponce , et al., “Is a Picture Worth a Thousand Words? A Scoping Review of the Impact of Visual Aids on Patients Undergoing Surgery,” Journal of Surgical Education 81, no. 9 (2024): 1276–1292: [Internet], 10.1016/j.jsurg.2024.06.002. 38955659

[29] P. Scalia , M. A. Durand , J. L. Berkowitz , et al., “The Impact and Utility of Encounter Patient Decision Aids: Systematic Review, Meta‐Analysis and Narrative Synthesis,” Patient Education and Counseling 102, no. 5 (2019): 817–841: [Internet], 10.1016/j.pec.2018.12.020. 30612829

[30] M. M. Becerra Pérez , M. Menear , J. C. Brehaut , and F. Légaré , “Extent and Predictors of Decision Regret About Health Care Decisions: A Systematic Review,” Medical Decision Making 36, no. 6 (2016): 777–790, 10.1177/0272989x16636113. 26975351

[31] M. M. Garvelink , L. Boland , K. Klein , et al., “Decisional Conflict Scale Use over 20 Years: The Anniversary Review,” Medical Decision Making 39, no. 4 (2019): 301–314, 10.1177/0272989x19851345. 31142194

[32] W. K. van Deen , B. M. R. Spiegel , and A. S. Ho , “A Narrative Review of Decision Aids for Low‐Risk Thyroid Cancer,” Annals of Thyroid 8, no. 3 (2023): 0–2, 10.21037/aot-22-9.

[33] M. J. Barry and S. Edgman‐Levitan , “Shared Decision Making—The Pinnacle of Patient‐Centered Care,” New England Journal of Medicine 366, no. 9 (2012): 780–781, 10.1056/nejmp1109283. 22375967

[34] G. Elwyn , D. Frosch , R. Thomson , et al., “Shared Decision Making: A Model for Clinical Practice,” Journal of General Internal Medicine 27, no. 10 (2012): 1361–1367, 10.1007/s11606-012-2077-6. 22618581 PMC3445676

[35] T. C. Hoffmann , F. Légaré , M. B. Simmons , et al., “Shared Decision Making: What Do Clinicians Need to Know and Why Should They Bother?,” Medical Journal of Australia 201, no. 1 (2014): 35–39, 10.5694/mja14.00002. 24999896

[36] S. Brodney , F. J. Fowler , M. J. Barry , Y. Chang , and K. Sepucha , “Comparison of Three Measures of Shared Decision Making: SDM Process_4, CollaboRATE, and SURE Scales,” Medical Decision Making 39, no. 6 (2019): 673–680, 10.1177/0272989x19855951. 31226911 PMC6791732

[37] F. R. Gärtner , H. Bomhof‐Roordink , I. P. Smith , I. Scholl , A. M. Stiggelbout , and A. H. Pieterse , “The Quality of Instruments to Assess the Process of Shared Decision Making: A Systematic Review,” PLoS One 13, no. 2 (2018): 1–57, 10.1371/journal.pone.0191747. PMC5813932 29447193

[38] F. Légaré and H. O. Witteman , “Shared Decision Making: Examining Key Elements and Barriers to Adoption into Routine Clinical Practice,” Health Affairs 32, no. 2 (2013): 276–284, 10.1377/hlthaff.2012.1078. 23381520

[39] S. M. Dy and T. S. Purnell , “Key Concepts Relevant to Quality of Complex and Shared Decision‐Making in Health Care: A Literature Review,” Social Science & Medicine 74, no. 4 (2012): 582–587, 10.1016/j.socscimed.2011.11.015. 22236643 PMC9723523

[40] S. Ahmadi , J. M. Gonzalez , M. Talbott , et al., “Patient Preferences Around Extent of Surgery in Low‐Risk Thyroid Cancer: A Discrete Choice Experiment,” Thyroid 30, no. 7 (2020): 1044–1052, 10.1089/thy.2019.0590. 32143553

[41] P. Dixon , G. Tomlinson , J. Pasternak , et al., “The Role of Disease Label in Patient Perceptions and Treatment Decisions in the Setting of Low‐Risk Malignant Neoplasms,” JAMA Oncology 5, no. 6 (2019): 817–823, 10.1001/jamaoncol.2019.0054. 30896738 PMC6567830

[42] B. Nickel , J. P. Brito , R. Moynihan , A. Barratt , S. Jordan , and K. McCaffery , “Patients’ Experiences of Diagnosis and Management of Papillary Thyroid Microcarcinoma: A Qualitative Study,” BMC Cancer 18, no. 1 (March 2018): 242, 10.1186/s12885-018-4152-9. 29499654 PMC5833084

[43] B. Nickel , K. Howard , J. P. Brito , A. Barratt , R. Moynihan , and K. McCaffery , “Association of Preferences for Papillary Thyroid Cancer Treatment With Disease Terminology: A Discrete Choice Experiment,” JAMA Otolaryngology–Head & Neck Surgery 144, no. 10 (October 2018): 887–896, 10.1001/jamaoto.2018.1694. 30140909 PMC6233835

[44] C. C. Lubitz , C. M. Kiernan , A. Toumi , et al., “Patient Perspectives on the Extent of Surgery and Radioactive Iodine Treatment for Low‐Risk Differentiated Thyroid Cancer,” Endocrine Practice 27, no. 5 (2021): 383–389, 10.1016/j.eprac.2021.01.005. 33840638 PMC10028733

[45] J. Hampton , G. Cooper , L. Wall , et al., “Risk of Cancer Recurrence Exerts the Strongest Influence Results of a Discrete Choice Experiment,” World Journal of Surgery 49, no. 5 (2025): 1254–1263, 10.1002/wjs.12520. 40044452 PMC12058448

[46] A. M. Sawka , S. Ghai , T. Yoannidis , et al., “A Prospective Mixed‐Methods Study of Decision‐Making on Surgery or Active Surveillance for Low‐Risk Papillary Thyroid Cancer,” Thyroid 30, no. 7 (2020): 999–1007, 10.1089/thy.2019.0592. 32126932 PMC7374636

[47] J. P. Brito , J. H. Moon , R. Zeuren , et al., “Thyroid Cancer Treatment Choice: A Pilot Study of a Tool to Facilitate Conversations With Patients With Papillary Microcarcinomas Considering Treatment Options,” Thyroid 28, no. 10 (2018): 1325–1331, 10.1089/thy.2018.0105. 29905089

[48] J. Wei , M. Thwin , B. Nickel , and A. Glover , “Factors Which Inform Individual Decision Making Between Active Surveillance, Hemithyroidectomy and Total Thyroidectomy for Low‐ Risk Thyroid Cancer: A Scoping Review,” Thyroid 32, no. 7 (2021): 807–818, 10.1089/thy.2021.0646. 35438545

[49] W. Yang , Y. K. Lee , P. Lorgelly , S. N. Rogers , and D. Kim , “Challenges of Shared Decision‐Making by Clinicians and Patients With Low‐Risk Differentiated Thyroid Cancer: A Systematic Review and Meta‐Ethnography,” JAMA Otolaryngology–Head & Neck Surgery 149, no. 5 (2023): 452–459, 10.1001/jamaoto.2023.0101. 36951823

[50] S. C. Pitt , M. C. Saucke , B. R. Roman , S. C. Alexander , and C. I. Voils , “The Influence of Emotions on Treatment Decisions About Low‐Risk Thyroid Cancer: A Qualitative Study,” Thyroid 31, no. 12 (2021): 1800–1807, 10.1089/thy.2021.0323. 34641715 PMC8721509

[51] J. Hampton , A. Alam , N. Zdenkowski , C. Rowe , E. Fradgley , and C. J. O’Neill , “Fear of Cancer Recurrence in Differentiated Thyroid Cancer Survivors: A Systematic Review,” Thyroid 34, no. 5 (2024): 541–558, 10.1089/thy.2023.0642. 38368547

[52] C. B. Jensen , M. C. Saucke , D. O. Francis , C. I. Voils , and S. C. Pitt , “From Overdiagnosis to Overtreatment of Low‐Risk Thyroid Cancer: A Thematic Analysis of Attitudes and Beliefs of Endocrinologists, Surgeons, and Patients,” Thyroid 30, no. 5 (2020): 696–703, 10.1089/thy.2019.0587. 31910092 PMC7232663

[53] C. B. Jensen , M. C. Saucke , and S. C. Pitt , “Active Surveillance for Thyroid Cancer: A Qualitative Study of Barriers and Facilitators to Implementation,” BMC Cancer 21, no. 1 (2021): 1–9, 10.1186/s12885-021-08230-8. 33910527 PMC8080390

[54] A. G. Antunez , M. C. Saucke , K. J. Bushaw , A. Chiu , and S. C. Pitt , “Surgeon Preference for Maximizing Medical Care Is Associated With Recommending More Extensive Surgery for Low‐Risk Thyroid Cancer,” Thyroid 34, no. 9 (2024): 1181–1185, 10.1089/thy.2024.0170. 39030827 PMC11958910

[55] J. M. Evron , D. Reyes‐Gastelum , M. Banerjee , et al., “Role of Patient Maximizing‐Minimizing Preferences in Thyroid Cancer Surveillance,” Journal of Clinical Oncology 37, no. 32 (2019): 3042–3049, 10.1200/jco.19.01411. 31573822 PMC6839910

[56] A. Doubleday , M. C. Saucke , M. F. Bates , and S. C. Pitt , “Patient‐Surgeon Decision‐Making About Treatment for Very Low‐Risk Thyroid Cancer,” Trends in Cancer Research 14, no. 1 (2019).

[57] C. J. O’Neill , M. A. Carlson , C. W. Rowe , E. A. Fradgley , and C. Paul , “Hearing the Voices of Australian Thyroid Cancer Survivors: Qualitative Thematic Analysis of Semistructured Interviews Identifies Unmet Support Needs,” Thyroid 33, no. 12 (2023): 1455–1464, 10.1089/thy.2023.0080. 37335225 PMC10734898

[58] A. Koot , R. Netea‐Maier , P. Ottevanger , R. Hermens , and P. Stalmeier , “Needs, Preferences, and Values During Different Treatment Decisions of Patients With Differentiated Thyroid Cancer,” Journal of Personalized Medicine 11, no. 7 (2021): 682, 10.3390/jpm11070682. 34357149 PMC8304194

