# Supplementary material for: Development of a Decision Aid for Patients With Low‐Risk Thyroid Cancer: A Mixed‐Methods Analysis of Feedback From Both Patient and Clinicians
Source: World J Surg. 2025 Aug 30;49(10):2782–93. doi: 10.1002/wjs.70064 (PMC12515032; doi:10.1002/wjs.70064)
Supplement: Supplementary file 1 — Supporting information S1 [file WJS-49-2782-s002.pdf]

Developing a decision aid for patients with low-risk thyroid cancer

Ahmad Alam, Elizabeth Fradgley, Christopher Rowe, Nick Zdenkowski, Christine O'Neill

John Hunter Hospital, NSW

There is robust evidence that shared decision making decreases decisional regret in cancer patients. However, there is little relevant patient information to assist with this process in low-risk thyroid cancer.

With surgery being the cornerstone for management of thyroid carcinoma, it is acceptable for many patients to choose either total thyroidectomy, thyroid lobectomy or active surveillance for their disease, with or without the need for radioactive iodine ablation. A surgical, oncology, and endocrinology team from Hunter New England Health and the University of Newcastle are currently developing written and online tools to assist clinicians and patients with shared decision making.We are seeking input from surgeons, oncologists, and endocrinologists on the value, content and format of a shared decision making tool. This includes feedback through this quick survey along with a seminars at RACS ASC 2022 on 4th May 2022 and the ESA Seminar Meeting on 30th April 2022. Our goal is to design a robust, practice-ready and pragmatic tool for Australian patients.Please complete the following survey so we can understand the diverse background and expertise of those interested in shared decision making. The survey is anonymous with the option to provide contact details.

A few questions about you and your practice

What is your gender?	◯ Female<br>◯ Male<br>◯ Other
Which state of territory is your main clinical practice?	◯ New South Wales<br>◯ Victoria<br>◯ Tasmania<br>◯ Queensland<br>◯ Western Australia<br>◯ South Australia<br>◯ Australian Capital Territory<br>◯ Northern Territory<br>◯ New Zealand<br>◯ Other
What is the state and country of your main practice location?	__________________________
What best describes your field of practice?<br><br>If you are in training, select the option that reflects your final practice field.	◯ Consultant Endocrinologist<br>◯ Consultant Endocrine Surgeon<br>◯ General Surgeon<br>◯ General Physician<br>◯ General Practitioner<br>◯ Cancer Coordinator Nurse<br>◯ JMO<br>◯ Other
Where are you up to in your career or training?	◯ 0-5 years post Fellowship<br>◯ 6-11 years post Fellowship<br>◯ 11-15 years post Fellowship<br>◯ >15 years post Fellowship<br>◯ Currently in a specialty training program (eg Endocrine AT, SET Trainee, GP registrar etc)<br>◯ Pre-vocational trainee (eg JMO)
Do you regularly participate in a Thyroid Cancer MDT?	◯ Yes<br>◯ No

Where do you see ambulatory patients with thyroid nodules or thyroid cancer?<br><br>Choose the best option	○ Public outpatient clinic<br>○ Private outpatient clinic<br>○ Both public and private outpatient clinics<br>○ I do not see ambulatory patients with thyroid nodules/thyroid cancer
Each year, how many different patients with thyroid cancer would you see ?<br><br>This includes new and follow up cases	○ < 10<br>○ 10-20<br>○ 20-50<br>○ >50
Each year, how many patients with with thyroid cancer would you see before their initial thyroid surgery?	○ < 10<br>○ 10-20<br>○ 20-50<br>○ >50

How do you currently discuss thyroid surgery with your patients for low risk thyroid cancer? This is a setting where guidelines do not offer a firm reccomendation regarding extent of surgery, and multiple factors can be considered in the optimum treatment decision.

For example, a middle aged patient with a 2cm PTC, and a modestly enlarged contralateral lobe containing several 10+mm TR3 nodules could reasonably be offered several surgical strategies (total thyroidectomy, hemithyroidectomy or even active surveillance). When we use the phrase "low risk thyroid cancer" we are considering a situation such as this.

If you do not ever discuss thyroid surgery with patients, please skip to the next section.

Do you use regularly use written information to communicate with patients regarding low-risk thyroid surgery?<br><br>Choose all options that apply.	☐ No, I just talk with them<br>☐ Yes, I draw a picture<br>☐ Yes, I use written material that I have written<br>☐ Yes, I use written material from another source<br>☐ Yes, I refer them to a website<br>☐ Other
Please tell us what external resources or websites you regularly provide to patients	__________________________________
In general in your practice, how would you say treatment decisions are made about low risk thyroid cancer surgery?	○ I recommend a treatment plan<br>○ The plan is shared equally between me and the patient<br>○ The patient is given the options and they make the decision
In your practice, how satisfied are you with the discussion around surgery for low risk thyroid cancer?	○ Very satisfied and no improvements are required ○ Somewhat satisfied, but could be improved ○ It is OK, but I would like to make changes. ○ This is an area of my practice that I would like to significantly improve<br>○ Very dissatisfied.
How would you like to improve your practice in this area?<br>Select all that apply	☐ The discussion goes for too long or is too complex ☐ Clearer explanation of treatment options ☐ Clearer explanation/documentation of risks<br>☐ I feel like the patient doesn't understand the options ☐ I feel the patient is pressured into following my recommendation<br>☐ I would like to involve the patient more in the decision making process<br>☐ Other
In what way would you like to improve your practice in this area?	__________________________________
In your opinion, do you think that patients in general would benefit from being more involved in treatment decisions?	○ Yes<br>○ No<br>○ Not sure

Your preferences regarding an ideal decision-aid If a decision aid were developed to assist with decision-making around the extent of surgery (eg total thyroidectomy, hemithyroidectomy or active surveillance) for low-risk thyroid cancer, what would be its ideal characteristics?

In your opinion, what settings would be appropriate for use of a decision aid?<br>Tick all that apply	☐ GP<br>☐ Endocrinologist<br>☐ Surgeon<br>☐ Endocrine Cancer Nurse<br>☐ Directly available to patients on a website
Would it be helpful to have standardised decision aid that is used by all parties?<br>Tick all that apply	☐ Yes, improves quality of communication<br>☐ Yes, empowers patients and caregivers to have informed decisions<br>☐ No, endocrinologists and surgeons present information differently<br>☐ No, each surgeon has their own practice and preference for presenting information
If a decision aid existed to discuss decision making in low-risk thyroid cancer, how likely are you to use it in your consultations?	◯ Use it in most consultations where it was relevant ◯ Use it selectively (depending on patient characteristics)<br>◯ Use it rarely/not at all
In your practice, when in the management journey would you use the decision aid in your setting?<br>Tick all that apply	☐ Before the initial consultation based on information from the referral<br>☐ During the consultation to structure the discussion about management<br>☐ In between two consultations to help patients ask appropriate questions<br>☐ I would not use a decision-aid
In your opinion, what is the best format for a decision aid in practice?<br><br>Tick all that apply	☐ Paper based (single page or booklet)<br>☐ Electronic based (website or apps)<br>☐ Combination of both
In your opinion, how would the use of a shared decision aid impact the length of consult?	◯ Shorten the consult<br>◯ Have no effect on the timing<br>◯ Lengthen the consult
In your opinion, what are the barriers to using a decision aid routinely in practice?<br><br>Tick all that apply	☐ Availability of decision aid for clinician to access<br>☐ Availability of decision aid for patient to access<br>☐ Culture of doctor-driven decision making<br>☐ Clinician concern about providing too much information<br>☐ Clinician concern about 'overwhelming the patient'<br>☐ Patient reluctance to participate in decision<br>☐ Lack of applicability to specific scenarios<br>☐ Lack of credibility of such tools<br>☐ Other
What other barriers do you identify?	__________________________________

Do you intend to attend or have you attended any of the following seminars?	☐ RACS ASC meeting at 7am on Wed 4th May 2022<br>☐ Breakfast Symposium - ESA Seminar at 7.30am on Sat 30th April 2022
Do you have any further comments?	____________________________________
Do you think that developing nationally standardized decision aids in other fields of thyroidology would be of value?	☐ No<br>☐ Yes, adjuvant radioactive iodine after low risk DTC<br>☐ Yes, treatment options for Graves' disease<br>☐ Yes, pre-conception management of hyperthyroidism<br>☐ Yes, other
What other decision aids do you think would be of value?	____________________________________
Please provide your email address if you would like to receive updates about this project	______________________________
