# Supplementary material for: Development of a Decision Aid for Patients With Low‐Risk Thyroid Cancer: A Mixed‐Methods Analysis of Feedback From Both Patient and Clinicians
Source: World J Surg. 2025 Aug 30;49(10):2782–93. doi: 10.1002/wjs.70064 (PMC12515032; doi:10.1002/wjs.70064)
Supplement: Supplementary file 2 — Supporting Information S2 [file WJS-49-2782-s005.pdf]

Participant Information Sheet

Deciding about surgery for people who may have low risk thyroid cancersVersion 1 Dated 01.04.23

About this survey

This survey has been developed as part of a research project from The University of Newcastle, and John Hunter Hospital, in Australia.

Who is this survey for?

This survey is to give feedback on a paper decision aid sheet that was given to you by your doctor whilst discussing surgery for thyroid nodules that may be cancerous.

What will I be asked to do?

After reviewing the sheet of paper you were given, you will be invited to answer a series of survey questions regarding that information. In addition, you will be invited to review a website that contains additional information, and provide further feedback. The surveys are designed to help us make the information more useful for people in your situation.

Who will see these results?

The survey results will only be reviewed by the research team. If you have any questions about your treatment, or wish to discuss any aspect of your clinical management, you must make an appointment with your treating doctor to discuss. The results of the survey from many users will be pooled together to improve the quality of information provided for future participants.

What is the benefit to me?

Survey responses that you provide will not influence your healthcare directly, and will not be communicated to your referring or treating clinician. Participating in this research may not directly benefit you, although the information provided may be useful in helping you understand your treatment options, to discuss with your doctor.

Who is supervising this research?

This research project has been approved by the Hunter New England Human Research Ethics Committee. Should you have any concerns about the conduct of this research, you can contact the Director of Research, John Hunter Hospital, on 02 4921 4140.

Do I have to participate?

Participation in this study is voluntary, and you can withdraw your consent to participate at any stage. Your decision whether or not to participate will not prejudice the care you receive from your treating team in any way. All study information is confidential, and is stored securely on research servers. Results of research will be presented in aggregate deidentified formats.

What do I do now?

If you are willing to provide feedback, please indicate your consent below.
If you would prefer not to participate, please close this website. if you would like to find out further information about your condition we suggest you discuss this with your General practitioner or
referring doctor.

Complaints about this research

Should you have concerns about your rights as a participant in this research, or you have a complaint about the manner in which the research is conducted, it may be given to the researcher, or, if an independent person is preferred, please contact the HNE Research Office, Hunter New England Local Health District, Level 3, POD, HMRI, Lot 1 Kookaburra Circuit, New Lambton Heights NSW 2305.

Telephone: 02 4921 4140. Email: HNELHD-ResearchOffice@health.nsw.gov.au

Thank you for taking time to consider this study.

Coordinating Principal Investigator/Chief Investigator   A/Prof Christine O'Neill, Hunter New England Health

Lead Coordinating Investigator Dr Ahmad Alam

Associate Investigators Dr Nicholas Zdenkowski, Dr Christopher Rowe, Dr Elizabeth Fradgley

Consent

Do you agree to take part in this research survey?

○ Yes
○ No

Thank you for your time. If you have any concerns and would like to discuss this project with someone you can speak to a member of the research team by contacting the Research Team, via email: HNELHD-SurgeryResearch@health.nsw.gov.au or phone 02 4923 6397. You may close the browser at any time.

Eligibility Questions

Please complete the questions below to determine your eligibility for this survey.

Are you 18 years or older?	◯ Yes<br>◯ No
Do you have a thyroid nodule where there is a chance it could be thyroid cancer?	◯ Yes<br>◯ No
Have you received a PAPER decision aid (see below) to help you decide about management options for your thyroid cancer by a clinician	◯ Yes<br>◯ No
You are eligible to complete this survey.<br>We would like to inform you that this survey consists of three parts. The first part is the survey you are about to attempt, which aims to gather your valuable feedback on the paper decision aid. After completing the first survey, you will be directed to the second part, which involves reviewing a website version of the decision aid. Lastly, we have a feedback survey on the website.<br>We value your participation and understand that your time is precious. Therefore, we are happy to offer a $30 gift card for completing all three parts of the survey.<br>In order to streamline the gift card distribution process, we kindly ask for your email and phone number.<br><br>Are you happy to continue?	◯ Yes<br>◯ No<br>◯ Yes, but continue at a later date OR on a different device
Mobile/telephone number	______________________________
Email address	______________________________

Thank you for taking the time to start this survey.

Although we really appreciate your time, you do not meet the eligibility criteria for this particular survey.

If you have any concerns and would like to discuss this project with someone you can speak to a member of the research team by contacting the research team, via email: HNELHD-SurgeryResearch@health.nsw.gov.au or phone 02 4923 6397.

You may close the browser at any time.

To complete this survey at a later date or on a different device (eg. iPad or computer), please click on the:

"Save & Return Later" button below. This will allow you to enter an email address, where a link will be sent to you. You can then continue the survey at any time on a device of your choice by clicking on the link sent to the email address provided.

Some questions about you

Before we ask you for your feedback, it would be helpful for us to understand a little about your circumstances and background.

Please complete the following questions.

What is your current age?

- ◯ 18 to 29
- ◯ 30 to 39
- ◯ 40 to 49
- ◯ 50 to 59
- ◯ 60 to 69
- ◯ 70+

What is your gender?

- ◯ Female
- ◯ Male
- ◯ Neither or prefer not to say

Do you identify as?

- ◯ Aboriginal
- ◯ Torres Strait Islander
- ◯ Both Aboriginal and Torres Strait Islander
- ◯ Neither
- ◯ Prefer not to say

If you would like the research team to arrange for an Aboriginal health worker to contact you to support you through this survey, you can speak to a member of the research team by contacting us on:

- Email: HNELHD-SurgeryResearch@health.nsw.gov.au

or

- Phone 02 4923 6397

What is your residential postcode?

____

Where did you see a specialist for your thyroid gland problem?

- ◯ Newcastle/Lake Macquarie
- ◯ Maitland
- ◯ Tamworth
- ◯ Port Macquarie
- ◯ Other

Please specify where?

____

What is your highest level of education?

- ◯ I did not complete high school
- ◯ I completed high school
- ◯ I completed a technical college certificate (eg TAFE) or a trade
- ◯ I completed university (bachelor's degree)
- ◯ I have completed further study after university (post-graduate degree)

How often do you need to seek help to understand health information from your doctor ?

- ◯ Never
- ◯ Rarely
- ◯ Sometimes
- ◯ Often
- ◯ Always

Where do you usually go to find out more information about your health?	◯ GP's or Specialists' office<br>◯ Online sources (websites, blogs, social media)<br>◯ Friends or family members<br>◯ Books or other printed materials<br>◯ Support groups or community organizations<br>◯ Other (please specify below)
Please specify	__________________________________
Who is the person you usually share your health concerns with after a specialist's appointment?	◯ General Practitioner (GP)<br>◯ Spouse or partner<br>◯ Other family member (e.g., parent, sibling, adult child)<br>◯ Friend<br>◯ Other (please specify below)<br>◯ No one, I prefer to keep my health concerns private.
Please specify	__________________________________

Survey for assessment of paper-based decision aid

The quesetions on this page relate to the paper decision aid that was provided at your appointment.

How easy or difficult was it to understand the information provided in the paper-based decision aid.<br><br>(The paper decision aid is the double-sided piece of paper which provides information about your treatment choices for thyroid cancer)	◯ Very difficult<br>◯ Fairly difficult<br>◯ Neither easy nor difficult<br>◯ Fairly easy<br>◯ Very easy
How comprehensive was the information presented in the paper-based decision aid provided?	◯ Minimal or no information of value to me<br>◯ Insufficient information<br>◯ The correct amount of information<br>◯ More information than I required<br>◯ An overwhelming amount of information
How long did you spend reviewing the paper-based decision aid during your consultation?	◯ No time<br>◯ < 5 minutes<br>◯ 5 minutes to 15 minutes<br>◯ 15 minutes to 30 minutes<br>◯ >30 minutes
How long did you spend reviewing the paper-based decision aid after the consultation?	◯ No time<br>◯ < 5 minutes<br>◯ 5 minutes to 15 minutes<br>◯ 15 minutes to 30 minutes<br>◯ >30 minutes
Please rate the layout and images of the paper-based decision aid. Also please add in any comments you have on it in the box below.	◯ Very poor<br>◯ Poor<br>◯ Average<br>◯ Good<br>◯ Excellent
Additional comments on images or layout of decision aid	________________________________
Did you find the personal area provided for jotting down your thoughts helpful?	◯ Not at all helpful<br>◯ Not very helpful<br>◯ Neutral<br>◯ Somewhat helpful<br>◯ Extremely helpful
How well was the decision aid explained within the context of the consultation?	◯ Not explained at all<br>◯ Poorly explained<br>◯ Adequately explained<br>◯ Somewhat well explained<br>◯ Very well explained
6. Did you think the decision aid favoured a particular treatment. If so, which one?	◯ Active surveillance<br>◯ Hemithyroidectomy<br>◯ Total thyroidectomy<br>◯ Well balanced
7. Do you have any other comments or feedback about the paper-based decision aid?	◯ Yes<br>◯ No

Please give us your feedback or comments here.

____

Thank you for providing feedback on the paper decision aid.

We have also developed a website which contains additional information. We would like you to review the website and tell us if it was useful.
- When you click 'submit', you will be directed to a web-based decision aid.
- Please click through the website and review the information. It may take between 5 and 15 minutes for you to review the information on the website.
- When you have finished looking at the website, please click "Give feedback" in the top right of the webpage to complete another survey.

Thank you for your time.
