# Supplementary material for: Development of a Decision Aid for Patients With Low‐Risk Thyroid Cancer: A Mixed‐Methods Analysis of Feedback From Both Patient and Clinicians
Source: World J Surg. 2025 Aug 30;49(10):2782–93. doi: 10.1002/wjs.70064 (PMC12515032; doi:10.1002/wjs.70064)
Supplement: Supplementary file 3 — Supporting Information S3 [file WJS-49-2782-s003.pdf]

Survey for assessment of web-based decision aid

Please choose the answer for each question that most accurately describes how you feel personally after having reviewed this patient decision aid.

In this survey we ask you to focus on the information presented on the website.

Please rate each section of the website in the following questions:

The information on treatment options for thyroid cancer

- ◯ Very poor
- ◯ Poor
- ◯ Average
- ◯ Good
- ◯ Excellent

The information on risks or side effects of each treatment option

- ◯ Very poor
- ◯ Poor
- ◯ Average
- ◯ Good
- ◯ Excellent

Specific aspects related to treatment options

- ◯ Very poor
- ◯ Poor
- ◯ Average
- ◯ Good
- ◯ Excellent

How helpful was the 'My preferences' section in helping you choose your decision?

- ◯ Counterproductive
- ◯ Unhelpful
- ◯ Undecided
- ◯ Somewhat helpful
- ◯ Extremely helpful

Was the amount of information in the web based decision aid ....

- ◯ Insufficient
- ◯ Barely enough
- ◯ Adequate
- ◯ Excessive
- ◯ Overwhelming

How would best describe the layout of the web based decision aid?

- ◯ Confusing and difficult to follow
- ◯ Somewhat disorganized
- ◯ Easy to navigate
- ◯ Well-organized and visually appealing
- ◯ Exceptional and engaging

Did you feel that the information provided in the web-based decision aid was easy to understand?

- ◯ Strongly disagree
- ◯ Somewhat disagree
- ◯ Neither agree nor disagree
- ◯ Somewhat agree
- ◯ Strongly agree

Did you find that the decision aid was used at the appropriate time in your decision making process? If not please explain why

- ◯ Yes
- ◯ No

Please state when would be a better time to use the decision aid

____

Which of the formats of the decision aid did you prefer?	◯ Paper based<br>◯ Web based<br>◯ Both formats equally
Based on your review of the paper and web based decision aid, which treatment option do you prefer	◯ Active surveillance<br>◯ Hemithyroidectomy<br>◯ Total thyroidectomy<br>◯ Unsure

Thank you for answering these questions so far and for reviewing both the paper and web-based material.

These final questions ask you about your treatment choices for thyroid cancer.

Regarding your thyroid nodule (or cancer).<br><br>Do you know which options are available to you?	◯ Yes<br>◯ No<br>◯ Unsure
Do you understand enough about each treatment option to be comfortable in making a decision?	◯ Yes<br>◯ No<br>◯ Unsure
Do you know the risks and side effects of each option?	◯ Yes<br>◯ No<br>◯ Unsure
Are you clear about benefits matter most to you?	◯ Yes<br>◯ No<br>◯ Unsure
Are you clear about which risks and side effects matter most to you?	◯ Yes<br>◯ No<br>◯ Unsure
Do you have enough support from others to make a choice?	◯ Yes<br>◯ No<br>◯ Unsure
Are you choosing a treatment option without pressure from others?	◯ Yes<br>◯ No<br>◯ Unsure
Do you have enough information to make a choice?	◯ Yes<br>◯ No<br>◯ Unsure
Are you clear about the best choice for you?	◯ Yes<br>◯ No<br>◯ Unsure
Do you feel sure about what to choose?	◯ Yes<br>◯ No<br>◯ Unsure

Please add any information about your choices and the information received if not covered by the questions above	__________________________________
Are there are any areas that you did not like about either the paper or website decision aid?<br><br>Please explain.	____
Do you have any suggestions to improve the decision aid?<br><br>Please explain.	____

We appreciate your participation in the survey. Your feedback will play a crucial role in the development of the decision aid.

We kindly request that you provide us with your email address and telephone number, as this will enable us to express our gratitude for your participation in the survey by offering you a $30 gift card.

Email address	__________________________________
Mobile/telephone number	__________________________________
To allow the voices of patients to be heard beyond survey data, we are planning a focus group session where you could freely express your views on the next version of this decision aid (once your suggestions and others have been incorporated).<br><br>This focus group will not be paid but a small token of thanks in the form of a $30 gift voucher will be provided and any costs covered. Would you be interested in participating in the focus group?	◯ Yes<br>◯ No
