# Supplementary material for: Development of a Decision Aid for Patients With Low‐Risk Thyroid Cancer: A Mixed‐Methods Analysis of Feedback From Both Patient and Clinicians
Source: World J Surg. 2025 Aug 30;49(10):2782–93. doi: 10.1002/wjs.70064 (PMC12515032; doi:10.1002/wjs.70064)
Supplement: Supplementary file 4 — Supporting Information S4 [file WJS-49-2782-s001.pdf]

Deciding about surgery for people who may have low-risk thyroid cancer

Thank you for your time and interest. As a clinician involved in treating thyroid cancer, we are asking for your input on two decision support resources:

A paper-based decision aid, designed to be used within the context of a consultation Review the paper based decision aid.pdf A web-based decision aid, designed to provide additional comprehensive information, to be used either before or after a consultation Review the website This suite of decision support resources has been developed in consultation with the Australian and New Zealand Endocrine Surgeons, and the Endocrine Society of Australia. They have undergone extensive alpha testing with clinicians and patients.

The decsison aids aim to assist patients and clinicians with shared decision making regarding extent of surgery for low-risk thyroid cancer.In situations where there are multiple options of management and patient preference of outcomes is paramount, shared decision making to achieve informed preferences is ideal. One way to promote shared decision making in low-risk thyroid cancer is to use well designed and structured decision aids.Patient decision aids have been shown to improve knowledge, reduce conflict in decision making and improve patient satisfaction with the decision-making process. We invite your feedback on these resources. Your feedback will be incorporated with patient feedback for further iterative testing.   Your participation is voluntary and anonymous, and your responses will be kept confidential. We appreciate your time and valuable input in helping us improve the quality of care for patients with thyroid cancer. At the end of these surveys, you will be invited to express interest in being involved in wider testing of these resources within clinical practice.

Coordinating Principal Investigator/Chief Investigator   A/Prof Christine O'NeillHunter New England Health and University of Newcastle

Associate Investigators   Dr Nicholas ZdenkowskiDr Christopher RoweDr Elizabeth FradgleyDr Ahmad Alam

Complaints about this researchShould you have concerns about your rights as a participant in this research, or you have a complaint about the manner in which the research is conducted, it may be given to the researcher, or, if an independent person is preferred, please contact the HNE Research Office, Hunter New England Local Health District, Level 3, POD, HMRI, Lot 1 Kookaburra Circuit, New Lambton HeightsNSW 2305. Telephone: 02 4921 4140. Email: HNELHD-ResearchOffice@health.nsw.gov.au and quote the reference number: 2023/ETH00819 EthicsThis research has been approved by the Hunter New England Human Research Ethics Committee of Hunter New England Local Health District, Reference 2023/ETH00819

Do you agree to take part in this research survey?

◯ Yes
◯ No

Thank you for your time. If you have any concerns and would like to discuss this project with someone you can speak to a member of the research team by contacting the Research Team, via email: HNELHD-SurgeryResearch@health.nsw.gov.au or phone 02 4923 6397. You may close the browser at any time.

Some questions about you

Before we ask you for your feedback, it would be helpful for us to understand a little about your circumstances and background.

Please complete the following questions.

What is your gender?	◯ Female<br>◯ Male<br>◯ Other<br>◯ Prefer not to say
Please specify your gender	______________________________
What is your age group?	◯ Under 30 years old<br>◯ 30-39 years old<br>◯ 40-49 years old<br>◯ 50-59 years old<br>◯ 60-69 years old<br>◯ 70 years or older
What is your practice setting?	◯ Public hospital<br>◯ Private practice<br>◯ Both public and private
What is the type of area where you practice?	◯ Metropolitan<br>◯ Regional<br>◯ Rural
How many years have you been in specialist practice?	◯ Less than 5 years post fellowship<br>◯ 5-10 years post fellowship<br>◯ 11-20 years post fellowship<br>◯ More than 20 years<br>◯ I am undertaking a fellowship or a registrar (in training)
How frequently do you consult with patients regarding thyroid nodule workup?	◯ Less than one case per month<br>◯ About 1-4 cases per month<br>◯ About 2-5 cases per week<br>◯ More than 5 cases per week
How many patients with thyroid cancer have you cared for in the last year?	◯ None<br>◯ 5 cases or less per year<br>◯ 6-12 cases per year (about one a month)<br>◯ 13-40 cases per year (about 2-3 per month)<br>◯ 41-100 cases per year (about 1-2 per week)<br>◯ More than 100 cases per year (more than 2 cases per week)
What is your speciality?	◯ Endocrine surgeon<br>◯ General Surgeon<br>◯ ENT Surgeon<br>◯ Endocrinologist<br>◯ Other
Please specify	______________________________

Practitioner Opinion Survey - Paper

This section covers feedback from the PAPER based version of the decision aid.

This is designed to be printed as a double-sided sheet of A4 paper which is given to the patient by the surgeon or endocrinologist within the consultation.

Helping you decide about treatment if you might have a low-risk thyroid cancer

Hunter Medical Research Institute

What treatment options may be available? *(there is more detail over the page)*

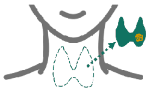

Total thyroidectomy
- The whole thyroid gland (including the cancer) is removed
- Thyroid hormone tablets must be taken lifelong
- There is a small chance of needing calcium tablets after surgery

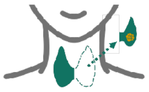

Hemithyroidectomy
- The half of the thyroid gland containing the cancer is removed
- Some people need to take thyroid hormone tablets after the surgery
- Calcium tablets are not needed
- The other half of the thyroid sometimes needs to be removed in the future

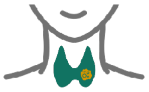

Active surveillance
- This is an option if the cancer is less than 10mm in size
- The cancer is not removed but is monitored with scans
- Surgery can be chosen later if your preferences change
- Surgery is recommended if the cancer grows
- This is not available at all centres and is only suitable for some people

**How do I feel about having surgery?**				
I would prefer to have my whole thyroid removed, and minimise the chances of another operation in the future	***or***	I would prefer to have a smaller operation first, minimising side effects but knowing there is a chance that I might need a second operation in the future	***or***	I would prefer to avoid surgery if possible
**How do I feel about the cancer coming back or growing?**				
I would prefer the cancer to be removed and I would do anything to decrease my chances of cancer returning	***or***	I would prefer the cancer to be removed now<br>I am happy that the remaining thyroid gland will need monitoring	***or***	I am happy to accept that the cancer is still present, and will be monitored closely
**How do I feel about taking tablets regularly?**				
I do not mind taking tablets for thyroid hormone or calcium every day		***or***		I would like to avoid taking tablets every day if possible

About "low-risk" thyroid cancers
- Low-risk thyroid cancers are small and have not grown outside the thyroid
- The chance of low-risk thyroid cancer coming back or spreading is less than 10%
- The chance of dying from low-risk thyroid cancer is close to zero

Deciding about treatment for low-risk thyroid cancer
- There are different treatment options that may be suitable
- The information here is to help you understand your choices
- This information could be used in discussion with your healthcare team and trusted friends or family
- Your surgeon or endocrinologist will work with you to provide clear advice on the best options for your treatment

These are some questions I have...

Note: The information in this decision aid is general information only and may not specifically apply to your situation. Risks and benefits listed are not exhaustive. It is not intended to form the basis of informed consent to a medical procedure. Detailed risks and benefits must be discussed with your doctor before commencing a course of therapy.

What are the main pros and cons for each treatment option?

	**Total thyroidectomy** 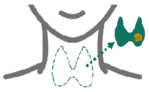	**Hemi-thyroidectomy** 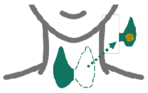	**Active surveillance** 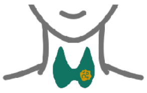
What is involved now?	*You have surgery to remove all of your thyroid gland*	*You have surgery to remove the half of your thyroid gland with cancer*	*You do not undergo any surgery unless the cancer grows over time*
What is involved over the next 5 years?	*After surgery you may have check-ups to ensure the cancer has not recurred*		*You will have check-ups every 6-12 months with ultrasound*
What are the benefits?	*You remove the cancer right away*		*Avoid surgery for a cancer that might never cause you harm*
What is my chance of dying of thyroid cancer in the next 10 years?	*Less than 1%*		
What is the chance of cancer growing or spreading in the next 5 years?	*Less than 5%*<br>*Recurrence can be detected with ultrasound or blood tests*	*5 -10%*<br>*Recurrence or growth can be detected with ultrasound*	
How long will I need to take off work initially?	*Most people return to work within 1-2 weeks of their operation*		*None (you do not have surgery)*
Will I need thyroid hormone tablets for the rest of my life?	*Yes, in all cases*	*30-50% chance of requiring*	*No*
What is the chance of needing calcium and vitamin D tablets for the rest of my life?	*2-5% chance*	*Close to zero*	*Zero*
What is the chance of major change in my voice volume or quality?	*2-4% chance*	*1-2% chance*	*Close to zero*
Will I need radioactive iodine?	*Radioactive iodine is not recommended for 'low-risk' cancers.*<br>*It can only be given if the whole thyroid has been removed*		

Please follow this link (or scan the QR code) to give feedback on this document: https://redcap.link/thyroid_aid

This aid has been developed by a HMRI/University of Newcastle research team, led by A/Prof C O'Neill. Version 3.01 19032024

The following questions asks about your perceptions of the PAPER-based decision aid (DA) and how you might use it in your clinical practice. Please indicate how strongly you agree or disagree with each statement

	Strongly disagree	Disagree	Neutral	Agree	Strongly agree
The DA will be easy for me to use	◯	◯	◯	◯	◯
The DA is easy for me to understand	◯	◯	◯	◯	◯
It will be easy for me to experiment with using the DA before making a final decision to adopt it	◯	◯	◯	◯	◯
The results of using the DA will be easy to see	◯	◯	◯	◯	◯
This DA is better than how I usually go about helping patients decide about extent of surgery	◯	◯	◯	◯	◯
This DA is compatible with the way I think things should be done	◯	◯	◯	◯	◯
Compared with my usual approach, this DA will result in my patients making more informed decision	◯	◯	◯	◯	◯
Using this DA will save me time	◯	◯	◯	◯	◯
This DA is a reliable method for helping patients make decisions about extent of surgery	◯	◯	◯	◯	◯
Pieces or components of this DA can be used by themselves	◯	◯	◯	◯	◯
This type of DA is suitable for helping patients make value-laden choices	◯	◯	◯	◯	◯
This DA complements my usual approach	◯	◯	◯	◯	◯
Using this DA will not require major changes to my usual practice	◯	◯	◯	◯	◯
Using this DA is likely to result in benefit, rather than harm, for my patients	◯	◯	◯	◯	◯

Did you think the decision aid was biased towards a particular treatment?

- ◯ Biased towards active surveillance
- ◯ Biased towards hemithyroidectomy
- ◯ Biased towards total thyroidectomy
- ◯ Well balanced (no bias)

Would you consider using this paper based decision aid in your practice?	◯ All patients where it was relevant<br>◯ Some patients where it was relevant<br>◯ I would not use this decision aid
What clinical scenarios would you consider the use of the paper decision aid appropriate?<br>Select all that apply	☐ Bethesda III nodules<br>☐ Bethesda IV nodules<br>☐ All Bethesda V/VI nodules<br>☐ Only Bethesda V/VI nodules that are likely to be low risk thyroid cancers<br>☐ Only in patients where I would consider less-than-total thyroidectomy<br>☐ I would not use this decision aid
What factors would make you consider using the paper decision aid with your patients?<br>Select all that apply	☐ Shorten the consultation<br>☐ Improve quality of information presented<br>☐ Improve shared decision making<br>☐ Reduce decision regret<br>☐ Patient requested more information<br>☐ Other
Please specify what other factors would influence you to use	__________________________________
What concerns would you have in using the paper decision aid?<br>Select all that apply	☐ Make the consultation longer<br>☐ Introduce unnecessary information<br>☐ Likelihood of making patient more anxious<br>☐ Likelihood of making patient more confused<br>☐ Using the DA doesn't fit with my style of practice<br>☐ Language or literacy barriers<br>☐ Other
Please specify your additional concerns	__________________________________
Please provide any general comments on the paper based decision aid<br><br>Should any information be added, removed or changed?	____

Practitioner Opinion Survey - Web

This section covers feedback from the WEBSITE decision aid.

The website is designed to be reviewed by the patient after the consultation, and used in conjunction with the paper based decision aid that you have already reviewed.

The website can take a few second to load. To best view and access all the website content, we strongly reccomend opening in a new window (click here)

The following questions asks about your perceptions of the WEBSITE decision aid and how you might use it in your clinical practice. Please indicate how strongly you agree or disagree with each statement

	Strongly disagree	Disagree	Neutral	Agree	Strongly agree
The wesbite will be easy for me to use	○	○	○	○	○
The website is easy for me to understand	○	○	○	○	○
It will be easy for me to experiment with using the website before making a final decision to adopt it	○	○	○	○	○
The results of using the website will be easy to see	○	○	○	○	○
This website is better than how I usually go about helping patients decide about extent of surgery	○	○	○	○	○
This website is compatible with the way I think things should be done	○	○	○	○	○
Compared with my usual approach, this website will result in my patients making more informed decision	○	○	○	○	○
Using this website will save me time	○	○	○	○	○
This website is a reliable method for helping patients make decisions about extent of surgery	○	○	○	○	○

Pieces or components of this website can be used by themselves	◯	◯	◯	◯	◯
This type of website is suitable for helping patients make value-laden choices	◯	◯	◯	◯	◯
This website complements my usual approach	◯	◯	◯	◯	◯
Using this website will not require major changes to my usual practice	◯	◯	◯	◯	◯
Using this website is likely to result in benefit, rather than harm, for my patients	◯	◯	◯	◯	◯

Did you think the website was biased towards a particular treatment?

- ◯ Biased towards active surveillance
- ◯ Biased towards hemithyroidectomy
- ◯ Biased towards total thyroidectomy
- ◯ Well balanced (no bias)

What factors would make you consider referring your patients to the website ?
Select all that apply

- ☐ Shorten the consultation
- ☐ Improve quality of information presented
- ☐ Improve shared decision making
- ☐ Reduce decision regret
- ☐ Patient requested more information
- ☐ Other

Please specify what other factors would influence you to use

____

What concerns would you have in referring your patients to the website?
Select all that apply

- ☐ Make the consultation longer
- ☐ Introduce unnecessary information
- ☐ Likelihood of making patient more anxious
- ☐ Likelihood of making patient more confused
- ☐ Using the DA doesn't fit with my style of practice
- ☐ Language or literacy barriers
- ☐ Other

Please specify your additional concerns

____

Please suggest any changes to the website content or layout

____

General feedback

Which format of the DA would you find most useful in your practice?	◯ Paper based only<br>◯ Web based only<br>◯ Both paper and web formats are potentially useful<br>◯ I would not use either format
When would you ideally like appropriate patients to first have access to the paper based DA?	◯ Before consultation with me<br>◯ During consultation with me<br>◯ After consultation with me<br>◯ Never
When would you ideally like appropriate patients to first have access to the website?	◯ Before consultation with me<br>◯ During consultation with me<br>◯ After consultation with me<br>◯ Never
Regarding the accessability of this website, I would prefer<br><br>(select all that apply)	☐ Link to the website from my own practice website<br>☐ Link to the website from a speciality society website<br>☐ Link to the website from a patient support group website<br>☐ Easily findable on search engines<br>☐ Difficult to access without direct link
Having reviewed this material, how likely would you be to use these decision aids in your clinical practice?	◯ Likely to use with most patients where it's relevant<br>◯ Use only with selected patients<br>◯ Unlikely to use
Please provide any final comments on these decision support resources	______________________________
Once final changes have been made to this decision aid we plan to run a multisite patient and clinician acceptability trial of the decision aid in clinical practice.<br><br>Would you be interested in participating in this trial or finding out more information?	◯ Yes<br>◯ No
Please provide your name	______________________________
Please provide your contact email address	______________________________

Thank you for taking the time to participate in our survey. Your input is invaluable in helping us gain insight to develop this decision aid. With your help, we hope to assist clinicians and patients with these decisions and improve patient satisfaction.
