# Supplementary material for: Development of a Decision Aid for Patients With Low‐Risk Thyroid Cancer: A Mixed‐Methods Analysis of Feedback From Both Patient and Clinicians
Source: World J Surg. 2025 Aug 30;49(10):2782–93. doi: 10.1002/wjs.70064 (PMC12515032; doi:10.1002/wjs.70064)
Supplement: Supplementary file 5 — Supporting Information S5 [file WJS-49-2782-s004.pdf]

Helping you decide about treatment if you might have a low-risk thyroid cancer

What treatment options may be available? *(there is more detail over the page)*

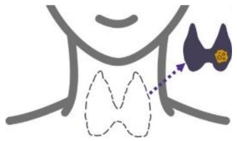

Total thyroidectomy

- The whole thyroid gland (including the cancer) is removed
- Thyroid hormone replacement tablets must be taken lifelong
- There is a small chance of needing lifelong calcium tablets after surgery

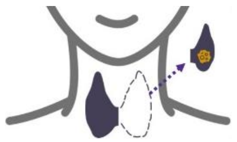

Hemithyroidectomy

- The half of the thyroid gland containing the cancer is removed. The other half is not removed, but is monitored over time with ultrasound scans.
- Some people need to take thyroid hormone tablets after the surgery
- Calcium tablets are not needed
- A second operation to remove the remaining thyroid is sometimes required

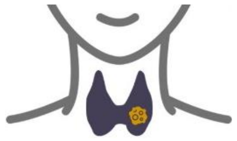

Active surveillance

- This is an option if the cancer is less than 10mm in size
- The cancer is not removed but is monitored with scans
- Surgery can be chosen later if your preferences change
- Surgery is recommended if the cancer grows

How do I feel about having surgery?

I would prefer to have my whole thyroid removed, and minimise the chances of another operation in the future	***or***	I would prefer to have a smaller operation first, minimising side effects but knowing there is a chance that I might need a second operation in the future	***or***	I would prefer to avoid surgery if possible

How do I feel about the cancer coming back or growing?

I would prefer the cancer to be removed and I would do anything to decrease my chances of cancer returning	***or***	I would prefer the cancer to be removed now<br>I am happy that the remaining thyroid gland will need monitoring	***or***	I am happy to accept that the cancer may not need to be removed, and will be monitored closely

How do I feel about taking tablets regularly?

I do not mind taking tablets for thyroid hormone or calcium every day	***or***	I would like to avoid taking tablets every day if possible

About "low-risk" thyroid cancers

- Low-risk thyroid cancers are small and have not grown outside the thyroid
- The chance of low-risk thyroid cancer coming back or spreading is less than 10%
- The chance of dying from low-risk thyroid cancer is close to zero

Deciding about treatment for low-risk thyroid cancer

- There are different treatment options that may be suitable
- The information here is to help you understand your choices
- This information could be used in discussion with your healthcare team and trusted friends or family
- Your surgeon or endocrinologist will work with you to provide clear advice on the best options for your treatment

These are some questions I have...

This aid has been developed by a HMRI research team, led by A/Prof C O'Neill. Version 4.21 04112024. *Note: The information in this decision aid is general information only and may not specifically apply to your situation. Risks and benefits listed are not exhaustive. It is not intended to form the basis of informed consent to a medical procedure. Detailed risks and benefits must be discussed with your doctor before commencing a course of therapy.*

What are the main pros and cons for each treatment option?

Hunter Medical Research Institute

	**Total thyroidectomy** 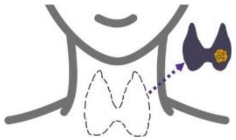	**Hemi-thyroidectomy** 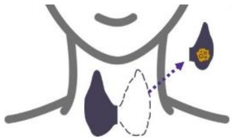	**Active surveillance** 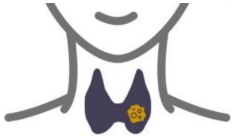
What is involved now?	*You have surgery to remove all of your thyroid gland*	*You have surgery to remove the half of your thyroid gland with cancer*	*You do not undergo any surgery unless the cancer grows over time*
What is involved over the next 5 years?	*After surgery you may have check-ups to ensure the cancer has not recurred*		*You will have check-ups every 6-12 months with ultrasound*
What are the benefits?	*You remove the cancer right away*		*Avoid surgery for a cancer that might never cause you harm*
What is my chance of dying of thyroid cancer in the next 10 years?	*Less than 1%*		
What is the chance of cancer growing or spreading in the next 5 years?	*Less than 5%*<br>*Recurrence can be detected with ultrasound or blood tests*	*5 -10%*<br>*Recurrence or growth can be detected with ultrasound*	
How long will I need to take off work initially?	*Most people return to work within 1-2 weeks of their surgery*		*None (you do not have surgery)*
Will I need thyroid hormone tablets for the rest of my life?	*Yes, in all cases*	*30-50% chance of requiring*	*No*
What is the chance of needing calcium and vitamin D tablets for the rest of my life?	*2-5% chance of permanent hypoparathyroidism*	*Close to zero*	*Zero*
What is the chance of major change in my voice volume or quality?	*2-4% chance*	*1-2% chance*	*Close to zero*
Will I need radioactive iodine?	*Radioactive iodine is not recommended for 'low-risk' cancers, but may be recommended if unexpected higher-risk features are found. It can only be given if the whole thyroid has been removed*		

If you would like more detailed information, please scan the QR code to visit this website
https://thyroidology.au/thyroid_aid
